# Plain Words on Registration.—V
*For previous articles see March 31, p. 521, April 7, p. 15, April 14, p. 35, and April 21, p. 53.


**Published:** 1917-05-12

**Authors:** 


### THE MATRONS' AND SISTERS' DEPARTMENT

### PLAIN WORDS ON REGISTRATION.—V.*

### The Central Committee's Registration Bill.

FROM A CORRESPONDENT.

The leaders of the party who describe themselves as Registrationists have from time to time expressed grave apprehension as to the baneful effect that V.A.D.s may produce when their services are no longer required by the military authorities. What is feared is that they may take up private practice and, so to speak, glut the market. The remedy for this evil, they declare, is State Registration. The State it was that called the V.A.D.s to active nursing duty, and, had the Nurses' Registration Bill, frequently presented to Parliament before the war, been placed on the Statute Book prior to 1914, it contained no provision which would have been an obstacle to the military authorities taking the action which they did, nor yet to the V.A.D.s either during or after the war embarking in private practice.

Registration can never be the panacea claimed for it, but it will differentiate between the fully-trained and the semi-trained. The medical profession cannot prevent the public being treated by quacks, but it can produce a Register of fully-qualified men so that the public can protect itself from the former if it desires. No harm is done by the action of the military authorities calling up a body of unskilled women to meet an emergency, but it is only fair to the State to give the public an opportunity of distinguishing between the skilled and unskilled when the emergency is past.

Parliamentary Bills are not, as a rule, easy reading to a mind untrained in law, but the provisions of the Nurses' Registration Bill are simple, and no member of the profession will experience any difficulty in comprehending their drift. May we suggest the propriety of a careful perusal! The Bill, published by the King's printers at the nominal cost of twopence, has not been revised or brought up to date. Copies of the latest revise may be obtained at 431 Oxford Street, London, W.

#### The Registrationist Creed.

The Nurses' Registration Bill may be regarded as a full confession of faith on the part of its supporters—*i.e.*, the Society for the State Registration of Trained Nurses. It would be superfluous to inquire further as to what they hope to achieve, inasmuch as this document is of their own manufacture. An Act sometimes contains tares sown by the spoiler, but there are no restrictions in the preparation of a Bill. "This Bill," runs the text, "has been prepared at the instance of the Central Committee for the State Registration of Nurses," and then the Memorandum proceeds to define what its promoters hoped to achieve by its enactment. "The object of the Bill is to ensure that the community shall have a guarantee that the nurses they employ are skilled in their professional duties, and through standardisation to give security to the public that the services of fully qualified nurses shall be readily obtainable." Wrapped up in these words is the prize which British Nurses have been persuaded contains their Salvation, and on which thousands of pounds, the earnings of hardworked women, are declared to have been expended. Translated into equivalent language, the words of the Bill signify that enrolment on a State Register is to be substituted for a Hospital-training Certificate—nothing more. Other Registration Acts are more far-reaching. The Medical Act, as already stated, earmarks a large number of appointments to be reserved for registered practitioners: it forbids the issue of certain certificates by unregistered persons: and, in addition, it renders impossible the recovery by legal process of fees for medical attendance by unregistered persons. Naturally it was to be expected that the Nurses' Registration Bill would make similar provision, but does not. Hence is apparent the hollowness of the claim that State Registration would prevent V.A.D. encroachment.

Having defined the authors and the objects of the Bill, its promoters proceed to the constitution and the powers of the Council which it proposes to establish, and to the method of electing representatives. The duties and powers of the Council are specified in some detail, but are definitely limited to "anything necessary for the due and proper carrying out the provisions of this Act." So that if this Council, when established, endeavoured by any means to improve the education, status, or conditions of service of British Nurses, they would be acting *ultra vires*.

#### Fees and Expenses.

Entrance to this land of promise is not to be free. In the most recent issue of the Nurses' Registration Bill no amount is specified, but in previous Bills it is provided that "there shall be payable by every candidate for examination or registration such fee as the Council may with the approval of the Privy Council from time to time determine, such fee not to exceed the sum of three guineas for examination, and two guineas for registration," and "there shall also be payable on or before the 31st January in each year by every registered nurse, a fee of two shillings and sixpence." Touching expenditure, it is provided that "there shall be paid to members of the Council such fees for attendance approved by the Privy Council, and such reasonable travelling expenses as shall from time to time be allowed by the Council."

Will our readers consider us guilty of any

* For previous articles see March 31, p. 521, April 7, p. 15, April 14, p. 35, and April 21, p. 53.

approach to bias or exaggeration in describing this beacon held up to guide the British Nurse as a Will-o'-the-wisp? It is indeed a lure-into-the-mist, and we cannot exempt from censure the faction responsible for the misdirection of the most honourable and hardworked profession of women in the world in so vital a matter. There are hundreds following blindly in the belief that only a hard and hostile world stands between them and happiness. For them we are profoundly sorry. Too busily occupied in fighting the battle of life, they should never put their trust in leaders with an axe to grind. Happily it is not yet too late for them to retrace their steps. Those who are wise will shape their course to secure their admission to the Register of the Royal College of British Nursing, quickly.

